# Adolescent seasonal allergic rhinitis and the impact of health-care professional training: cluster randomised controlled trial of a complex intervention in primary care

**DOI:** 10.1038/npjpcrm.2014.12

**Published:** 2014-06-05

**Authors:** Victoria S Hammersley, Rob A Elton, Samantha Walker, Christian H Hansen, Aziz Sheikh

**Affiliations:** 1 Allergy and Respiratory Research Group, Centre for Population Health Sciences, The University of Edinburgh, Edinburgh, UK; 2 School of Molecular and Clinical Medicine, The University of Edinburgh, Western General Hospital, Edinburgh, UK; 3 Division of General Internal Medicine and Primary Care, Brigham and Women’s Hospital, Harvard Medical School, Boston, MA, USA

## Abstract

**Background:**

Seasonal allergic rhinitis is typically poorly managed, particularly in adolescents, in whom it is responsible for considerable morbidity. Our previous work has demonstrated that if poorly controlled this can impair educational performance.

**Aim:**

The primary aim of this trial was to assess the impact of a primary care–based professional training intervention on clinical outcomes in adolescents with seasonal allergic rhinitis.

**Methods:**

Cluster trial in which UK general practice staff were randomised to a short, intensive workshop on the evidence-based management of seasonal allergic rhinitis. The primary outcome measure was the change in the validated Rhinoconjunctivitis Quality of Life Questionnaire with Standardized Activities (RQLQ(S)) score between baseline and 6 weeks post intervention (minimal clinically important difference=0.5). Secondary outcome measures of interest included health-care professionals’ knowledge and confidence in managing seasonal allergic rhinitis, number of seasonal allergic rhinitis-related consultations, relevant treatments prescribed and symptom scores.

**Results:**

Thirty-eight general practices were randomised (20 in the intervention arm) and 246 patients (50.2% males, mean age 15 years) were included in the primary outcome analysis. Health-care professionals’ knowledge and confidence of the clinical management of seasonal allergic rhinitis improved. This did not, however, result in clinically or statistically significant improvements in RQLQ(S): −0.15, (95% confidence interval, −0.5 to +0.2). There were no differences in consultation frequency, treatments issued for seasonal allergic rhinitis or symptom scores.

**Conclusions:**

Although associated with increases in professionals’ self-assessed confidence and understanding of seasonal allergic rhinitis management, this intensive training workshop did not translate into improvements in adolescents’ disease-specific quality of life or a reduction in rhinitis symptoms.

## Introduction

Seasonal allergic rhinitis (also known as intermittent allergic rhinitis) is a very common condition in adolescence, with national^1^ and international^2–4^ studies suggesting that up to 40% of young people may be affected. It can be responsible for considerable morbidity in its own right; research focusing on children and adolescents with seasonal allergic rhinitis has identified particular problems with schoolwork,^5^ exam performance^6^ as well as loss of sleep and reduced ability to concentrate.^7,8^ As a consequence, seasonal allergic rhinitis poses a substantial and increasing economic burden on health-care systems and the society at large.^9^ American estimates have, for example, suggested that health-care expenditure associated with allergic rhinitis has doubled since 2000, increasing to more than $11 billion.^10^ It is now better recognised that many people with seasonal allergic rhinitis also have coexistent asthma and this, together with a greater appreciation of their shared pathophysiology, has led the World Health Organization to promote the idea of ‘one airway, one disease’—i.e., that allergic rhinitis and asthma are different manifestations of the same disease.

Considerable time and resources are expended on educational interventions for health-care professionals, both in the United Kingdom and internationally, but despite these investments their impact on patient outcomes is still unknown. This is because such interventions are rarely evaluated beyond simple measures of satisfaction completed by the attendee, with little or no assessment of whether there is any impact on clinical practice or benefits to patients. Any intervention requiring time and/or financial commitment should be subject to the same rigorous evaluation as any other pharmacological or non-pharmacological intervention, and this is particularly true in financially constrained times. More specifically, the management of people with seasonal allergic rhinitis (and allergic rhinitis more generally) has been highlighted as being suboptimal by a number of studies^11,12^ and guidelines,^8^ with these inadequacies resulting in substantial—potentially avoidable—morbidity and cost. The overwhelming majority of people with seasonal allergic rhinitis are managed in the community;^13^ hence, this sector needs to be the focus of any attempt to improve the quality of care and outcomes.^2^

In an earlier multicentre randomised controlled trial,^14^ it was demonstrated that a part-time 6-month diploma-level allergy course was acceptable to attending primary health-care professionals, led to changes in relevant process measures (such as knowledge and confidence) and, importantly, translated into significant improvements in validated measures of disease-specific quality of life in adults. Although effective, many primary health-care professionals found this length of training difficult to incorporate into their practice, which raises important questions about the wider generalisability and sustainability of this approach. Following the approach advocated by the Medical Research Council’s Framework for Complex Interventions,^15,16^ we used our experiences from this earlier trial and related work on the training needs of primary care professionals in the context of managing seasonal allergic rhinitis to inform the development of the current intervention.^12,17,18^ In seeking to mirror the ways in which the majority of UK health-care professionals receive their continuing professional education, we developed an intensive, evidence-based 1-day educational training intervention for primary care professionals. We then sought to evaluate the effectiveness of this training intervention for primary care–based health-care professionals on adolescent disease-specific quality of life.

## Materials and Methods

### Overview of study design

We conducted a cluster randomised controlled trial of an educational intervention, which involved randomising general practice staff to receive either evidence-based allergy training or to the control arm in which practices received written guidance on the evidence-based management of seasonal allergic rhinitis.^19^ We chose a cluster randomised controlled trial, as a parallel group trial design would have resulted in a high risk of contamination. In keeping with recommended practice, our trial protocol and detailed analysis plan were reported before the closure of the trial;^20^ any deviations from the methods described in the protocol are detailed below.

### Setting and participants

This trial took place during the summers of 2009 and 2010 in 38 general practices within the recruitment areas of the Scottish Primary Care Research Network and England’s Northern and Yorkshire Research Network. Scottish Primary Care Research Network and England’s Northern and Yorkshire Research Network invited general practices to take part by email and letter. Each consenting practice was asked to nominate a member of their team to participate who had not received postgraduate allergy training in the previous 12 months. All patients aged 12–18 years with current seasonal allergic rhinitis were eligible to participate. We defined current seasonal allergic rhinitis by the presence of a documented clinician diagnosis in the patient’s electronic health record and any evidence of treatment(s) used for seasonal allergic rhinitis in the last 2 years.^21^ Patients who fulfilled these criteria were invited to take part in the trial via a letter from the practice, which included a participant information sheet, consent form and reply envelope for return directly to the research team.

### Randomisation and blinding

Practices were stratified on the basis of each of six regions: NHS Lothian; NHS South of Tyne and Wear; NHS North of Tyne; NHS County Durham; NHS North Yorkshire and York; and NHS Leeds; for regions where there were more than two clusters, a centrally administered minimisation scheme^22^ based on practice size and deprivation score was applied. Patients were masked to the allocation; however, it was not possible to mask the general practices as the intervention was attendance at a training workshop.

### Intervention

Education for Health (http://www.educationforhealth.org.uk/) is one of UK’s leading independent national training organisations offering accredited allergy training for professionals. We worked with Education for Health to develop an intensive 1-day evidence-based workshop, which was customised to meeting the needs of adolescents (see Box 1). The intervention consisted of an intensive study day focused on the diagnosis and management of young people with seasonal allergic rhinitis. The programme began with an assessment of the participant’s current allergy knowledge, followed by the presentation of a case study, a discussion about the importance of getting the diagnosis of allergy correct and reasons for treatment failure. This was then followed by a brief overview of the pathophysiology of allergy in general, before moving into a more detailed discussion of this in the context of seasonal allergic rhinitis and asthma. Embedded within the programme were practical sessions on nasal spray and inhaler device technique. Delegates were given a copy of the British Society of Allergy & Clinical Immunology allergic rhinitis algorithm,^19^ and all treatment discussions were based on the World Health Organization’s Allergic Rhinitis and Asthma^23^ and British Thoracic Society/Scottish Intercollegiate Guidelines Network (http://www.sign.ac.uk/guidelines/fulltext/101/index.html) guidelines on the management of allergic rhinitis and asthma, respectively (current at the time of the study). There was ample time for individual and group discussions to ensure that individual learning styles were catered for and any queries were addressed.

Health-care professionals (1 general practitioner and 19 practice nurses) nominated by the general practices who were randomised to the intervention arm attended these 1-day intensive workshops delivered by experienced Education for Health trainers. The course was repeated on five occasions.

### Control group

Health-care professionals (again nominated by the practices and in this case all nurses) in the control practices received an allergic rhinitis algorithm developed by the British Society of Allergy & Clinical Immunology,^19^ which was adapted for use in primary care by Education for Health. No training was offered to the 18 health-care professionals randomised to the control arm.

### Objectives and outcomes

Our primary outcome of interest was the difference in the change in adolescents Standardised Rhinoconjunctivitis Quality of Life Questionnaire (RQLQ(S)) score between baseline and 6 weeks post intervention in the intervention arm compared with the control arm. Consenting patients completed the RQLQ(S) and a symptoms score, which was measured on a 10-point visual analogue scale. Data were collected from two cohorts of patients in two separate years: 2009 and 2010. Baseline RQLQ(S) and symptom scores were recorded in May and early June 2009/2010; patients were then seen by their health-care professional, and follow-up RQLQ(S) and symptom scores were recorded in late June and July 2009/2010 (i.e., during the peak of the grass pollen season).

Our secondary outcomes of interest were:

- Assessment of change in clinical practice among the health-care professionals allocated to the intervention arm: the effects of the 1-day training on self-reported professional confidence, and understanding and clinical management of seasonal allergic rhinitis, which were measured immediately before and after the training days, and after all the patients taking part in the study had been seen by a health-care professional (~7–28 days), using a questionnaire that incorporated a 5-point Likert scale (1=less confident and 5=more confident).
- Total number of consultations for seasonal allergic rhinitis.
- Total number of prescribed medications for seasonal allergic rhinitis.
- Patient-reported symptom scores.

### Pollen data

Grass pollen data from York and Edinburgh (which covered the areas from which practices were drawn) were centrally provided by the National Pollen and Aerobiology Research Unit for 2009 and 2010 in order to assess whether the pollen counts reached a level that was likely to trigger seasonal allergic rhinitis symptoms during the study period. The pollen count is a measure of the number of pollen grains per cubic metre (pg/m^3^) of air sampled, averaged over 24 h.

### Sample size calculations

We calculated that a target sample size of 220 would give 80% power to detect a mean difference of 0·5 in RQLQ(S) score—the minimal clinically important difference at the 5% significance level. This was calculated using Sampsize^24^ assuming an s.d. of 1.2^25^ and intraclass correlation (ICC) of 0.02. There is little evidence in the literature of the likely size of the design effect from clustering in trials of this kind, and the choice of the relatively low ICC value of 0.02 was based mainly on the finding of no evidence of clustering in our earlier adult study using RQLQ.^14^

### Statistical analysis

We undertook a complete case analysis for our main analysis. In the primary analysis, multilevel modelling using a random effects model was used to take account of between- and within-cluster variation, adjusting for strata, individual covariates and year of study. Estimates and confidence intervals of the intervention effects are reported for the RQLQ(S) and symptom score.

Consultation and prescribing data were collected from the participating general practices for all patients from the date of consultation for the study to 31 August 2009/2010. Differences between the two groups were analysed using multilevel analysis in MLWin (version 2.20, 2010, http://www.bristol.ac.uk/cmm/software/mlwin/).

Differences in the mean scores of self-reported confidence and understanding of seasonal allergic rhinitis management in the intervention group were compared using *t*-statistics.

We first used mixed-model analysis of variance (ANOVA) using SPSS (version 14, 2005, SPSS, Chicago, IL, USA) to estimate the ICC for RQLQ(S), followed by a Bayesian approach using WinBUGS (version 1.4, 2009, http://www2.mrc-bsu.cam.ac.uk/bugs/winbugs/contents.shtml), taking a uniform prior over 0–1.^26^

### Deviations from the trial protocol

#### Educational data

We were unable to collect educational data because of time constraints resulting from the delays in recruiting practices.

#### Follow-up RQLQ(S)

Follow-up primary outcome data were collected by post and the majority were collected 6 weeks post intervention as planned and described in the trial protocol;^20^ however, there were a small number of questionnaires collected between 6 and 8 weeks post intervention as a result of non-responders to the initial mailing and the need to issue reminders.

#### Missing data

Our complete case analysis provided unbiased estimates under the assumption that the missing data are ‘Missing Completely At Random’;^27^ however, analysis of the means estimated separately for each pattern of missingness suggested that this assumption may not hold. We therefore carried out further sensitivity analyses testing a variety of assumptions:

- The direct likelihood method:^28^ the primary analysis of the effect of the intervention on RQLQ(S) was repeated, but with the baseline score modelled jointly with the outcome at 6 weeks instead of entering the model through the linear predictor. This model allowed for the inclusion of all 309 patients with data on at least one occasion and provided likelihood-based estimates that are valid under ‘Missing At Random’^27^ (under the assumption that the responses are multivariate normal).
- Multiple imputation: Proc MI in SAS (SAS Institute, Cary, NC, USA) was used to generate multiple imputations separately for each treatment arm (pooling across centres) (*m*=100 imputations).
- Alternative scenarios under ‘Missing Not At Random’^27^ for poor and good outcomes: first, under a poor outcome assumption, we imputed missing values in any particular cluster (at baseline or follow-up) using the largest observed score from that cluster (and time point). Second, under a good outcome assumption, we used the lowest observed scores from each cluster (and time point) to impute missing values.

## Results

### Baseline characteristics

**Figure 1:**
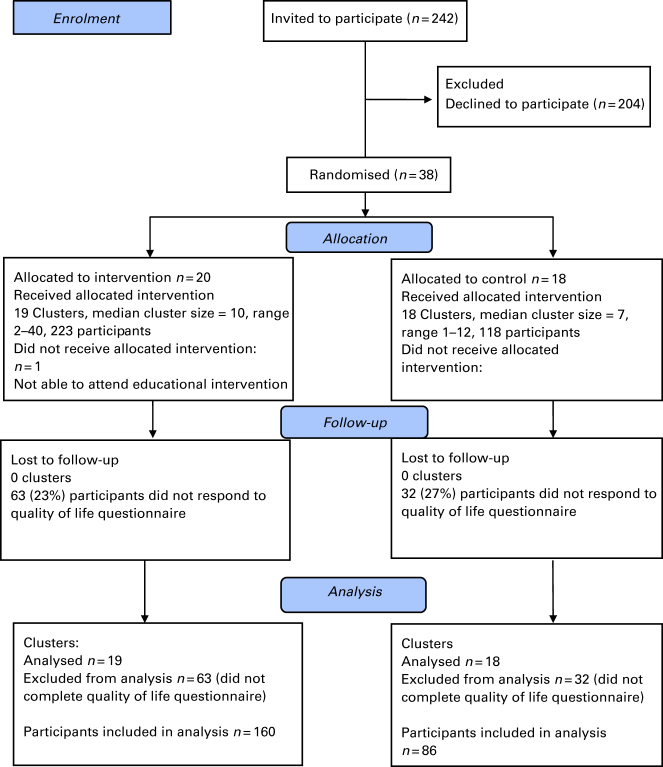
Flow of clusters and patients through the trial.

Thirty-eight general practices (clusters) agreed to participate in the study, of which 20 were randomised to the intervention arm and 18 to the control arm. Of the patients assessed for eligibility from the general practice medical records, 1,565 satisfied our inclusion criteria and, of them, 341 agreed to participate (see Figure 1).

Participating (*n*=38) and non-participating practices (*n*=204) had comparable demographic characteristics. In Scottish sites, the mean and s.d. of list size for participating practices was 6,562 (3,363) and that for non-participating practices was 6,971 (3,302) (*P*=0.75); the mean deprivation quintiles were 2.69 (s.d. 0.67) and 2.60 (s.d. 0.70) (*P*=0.76), respectively. In English sites, the mean list size for participating practices was 8,707 (s.d. 4,048) and for non-participating practices 7,464 (s.d. 4,847) (*P*=0.19); the Index of Multiple Deprivation scores were 26.8 (s.d. 18.2) and 31.0 (s.d. 19.6) (*P*=0.27), respectively.

Clusters were comparable for baseline characteristics in terms of deprivation; however, the intervention practices had a larger mean list size (see Table 1). Participants were comparable at baseline in terms of age and sex profiles.

### Primary outcome: impact on seasonal allergic rhinitis quality of life

A total of 246/341 patients (50·2% male, mean age 15 years) were included in the primary outcome analysis. The intervention failed to result in a clinically important improvement in RQLQ(S) (−0.15, 95% confidence interval (CI), −0.52 to +0.21) (adjusted for baseline RQLQ(S)), practice list size and region, year of study and deprivation.^29^

### Secondary outcomes

#### Assessment of change in clinical practice

Health-care professionals’ self-assessment of their confidence and understanding of seasonal allergic rhinitis management markedly increased post intervention when compared with the baseline assessment (see Table 2). All scores improved from Time 1 (immediately before the training day) to both Time 2 (immediately after the training day) and Time 3 (after all patients had been seen as part of the study).

#### Consultation and prescribing data

Five of the 38 practices did not provide data on consultation and prescribing patterns (three control and two intervention arm practices). Table 3 summarises data revealing that the intervention arm practices tended to have more consultations and prescriptions in total, and also more consultations for other respiratory conditions, but that the figures for seasonal allergic rhinitis did not differ greatly between the two arms.

#### Grass pollen and symptom score data

**Figure 2:**
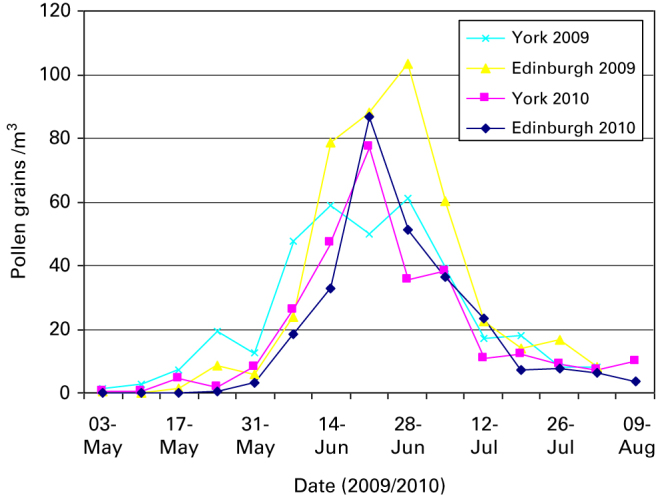
Pollen count in 2009/2010. Notes: The pollen forecast is usually given as low (<30 pg/m^3^), moderate (30–49 pg/m^3^), high (50–149 pg/m^3^) or very high (150 pg/m^3^). pg, pollen grains

Figure 2 indicates that the grass pollen reached sufficiently high counts (50–149 pg/m^3^) at both sites in both years to induce seasonal allergic rhinitis symptoms and that there was no significant regional variation between the two collection sites. Adjusted symptom scores in the intervention group were slightly lower than in the control group (−0.24, 95% CI, −1.03 to +0.54).

### Estimates of ICC

The ICC was estimated as 0.034 after adjusting for baseline RQLQ and the intervention group, with a 95% credible interval of 0.0016–0.145.

### Sensitivity analysis for impact on quality of life

By using the direct likelihood method^28^ to account for the missing data under a Missing At Random mechanism, the (adjusted) effect of the novel intervention on RQLQ(S) at 6 weeks was found to be 0.03 (95% CI, −0.33 to 0.39), supporting the finding that there is no beneficial effect of the intervention on RQLQ(S) score.

We obtained similar results with multiple imputation methods based on 100 imputed data sets. Using multiple imputations to account for the missing data, the intervention effect was 0.06 (95% CI, −0.30 to 0.42).

Finally, under the poor outcomes scenario, imputing missing data using large RQLQ(S) values, the estimated effect of the intervention was 0.21 (95% CI, −0.21 to 0.63). Under the good outcomes scenario, using low RQLQ(S) values to impute the missing data, the estimated intervention effect was 0.03 (95% CI, −0.39 to 0.44). The conclusions were thus unchanged: the intervention failed to have the desired effect.

## Discussion

### Main findings

This large general practice–based cluster randomised controlled trial has shown that a short intensive evidence-based allergy workshop for health-care professionals led to substantial and persistent improvements in their self-reported confidence and understanding of the management of seasonal allergic rhinitis, but this did not translate into changes in clinical practice in terms of frequency of consultations or prescribing habits; most importantly, this did not lead to clinically significant improvements in disease-specific quality of life or symptom score in adolescents with seasonal allergic rhinitis.

### Strengths and limitations of this study

The main strength of this trial was the decision formally to evaluate this evidence-based educational intervention using an adequately powered cluster randomised controlled trial, which is most uncommon. We developed a complex intervention based on a previous effective educational intervention for health-care professionals and measured the effectiveness of this on a validated disease-specific quality of life measure; in keeping with the Medical Research Council’s complex intervention framework,^16^ we also measured a range of relevant process measures, which aimed to shed light on the mechanisms through which any changes were mediated and/or blocked. We have demonstrated the acceptability of the intervention and that it has an impact on professionals’ self-assessed confidence and knowledge of seasonal allergic rhinitis management, but that the intervention did not equip individuals with the ability to enact relevant structural changes in their practices to translate this into improvements in care processes.

We conducted a complete case analysis supplemented by sensitivity analyses to assess the likely impact of the missing data. Our sensitivity analyses strengthened our finding of no beneficial effect of the intervention on RQLQ(S). A limitation of this study may be that patients in the control arm of the cluster trial also consulted with a health-care professional, which it could be argued may not reflect routine primary care. It was necessary to design the study this way in order to understand the cause of any potential effectiveness of the educational intervention and to be able to distinguish this from any impact of simply being seen for seasonal allergic rhinitis. Control arm practices received an algorithm and information leaflet for the management of seasonal allergic rhinitis, both of which were adapted by Education for Health. One way of disentangling this issue would have been to include a third arm in which practices received no intervention; however, this was not possible within the constraints of this trial. An additional possible limitation of this study is that cluster sizes were not balanced. We used general practice list size in the minimisation scheme; we may, however, have reached a better balance in clusters if we had used number of adolescents, as this varied between clusters more than we had anticipated. The effect of this imbalance on the power of the study was small, and we still achieved the power required.

### Interpretation of findings in relation to previously published work

Patients in this study had on average relatively mild impairment of quality of life measured by the RQLQ(S), which is in line with a similar study exploring the quality of life of perennial rhinitis sufferers.^14^ Patients were recruited from a primary care setting, using a clinician diagnosis of seasonal allergic rhinitis or a prescription for drugs used in nasal allergy in the last 2 years, rather than objective evidence of moderate or severe disease. As also observed in our earlier perennial rhinitis trial,^14^ the impact of this training intervention may have been more evident if we had restricted trial entry to those with more severe disease. This would, however, have reduced the generalisability of the intervention to everyday general practice.

We would have expected more consultations in the intervention group if the training had achieved a sustained change in clinical practice, but this was not the case. The training days were delivered by practising health-care professionals and based on current evidence-based guidelines for the management of seasonal allergic rhinitis developed by the British Society of Allergy & Clinical Immunology.^19^ The prescribing data included all repeat prescriptions; therefore, if the intervention practices followed the guidance given in the workshop, patients with persistent symptoms would be receiving an antihistamine, nasal steroid and/or topical ocular treatments, as appropriate.

### Implications for future research, policy and practice

Future trials need to build on the findings of both this and our earlier trial,^14^ and find ways of equipping participants of such short courses with the skills necessary to bridge the gap between knowledge and day-to-day practice. A key consideration is not only to upskill primary care–based health-care professionals but also to develop their ability to effect organisational change. The increasing opportunities for blended learning—i.e., a combination of both face-to-face and virtual training—should also provide opportunities for periodic, convenient and accessible reinforcement of key messages. Developing such initiatives and then formally trialling their effectiveness is important to help ensure that the National Health Service and other health-care systems internationally invest their limited resources in evidence-based educational interventions of proven effectiveness.

### Conclusions

In conclusion, this intensive seasonal allergic rhinitis training workshop for primary care health-care professionals was found acceptable and increased self-assessed confidence in attendees, but this did not translate into improvements in symptom control or quality of life of adolescents with seasonal allergic rhinitis.

## Figures and Tables

**Table 1:** Baseline information for each group at individual and cluster level

	*Intervention arm*	*Control arm*
*Practice factors*		
Number of clusters	20	18
Mean list size	11,144	8,330
Mean deprivation score		
IMD^a^	21.5	21.7
SIMD^b^	2.48	2.47
		
*Patient factors*		
Number	223	118
Mean age (years) (s.d.)	15 (1.85)	15 (1.91)
Number (%) male	112 (50.2)	57 (48.3)

a IMD—The 2004 Index of Multiple Deprivation for English practices.

b SIMD—Scottish Index of Multiple Deprivation.

**Table 2:** Audit of confidence in delivering allergy care (*n*=21)

*Question*	*Time 1* a	*Time 2* a	*Time 3* a	*95% CI* b
*How confident are you at*				
Taking a comprehensive allergy history from a patient with suspected allergy?	2.6	4.2	4.4	1.0, 2.2
Doing skin prick testing?	1.1	2.7	2.4	0.4, 2.6
Ordering specific IgE test?	1.5	3.8	3.5	1.2, 2.8
Making a diagnosis of allergy?	2.1	4.4	4.3	1.6, 2.7
Explaining the various effective treatment strategies for allergic problems?	2.2	4.3	4.7	1.9, 2.9
Prescribing/recommending treatment for allergic conditions?	2.3	4.1	4.6	1.4, 2.8
Teaching patients how to use nasal spray devices?	2.3	4.7	5.0	1.9, 3.2
Explaining the causes and mechanisms of allergy?	2.4	4.2	4.5	1.4, 2.7
Understanding the impact of allergy on morbidity and mortality?	2.6	4.2	4.5	1.4, 2.7
				
*How likely are you to do the following*				
Ask about other allergic symptoms (e.g., nose/skin) when assessing a patient with asthma?	3.3	4.8	4.8	0.6, 2.0
Consider total steroid use in patients on multiple therapies?	2.6	4.2	4.1	1.0, 2.2
Offer practical advice on avoiding allergens?	3.1	4.8	4.9	1.0, 2.4
Suggest patients use their nasal steroids regularly?	3.1	4.9	5.0	1.3, 2.5

Abbreviation: CI, confidence interval.

a Time 1—immediately before the training day, Time 2—immediately after the training day, Time 3—after all patients had been seen as part of the study (range 7–28 days).

b 95% CI for change in mean score from Time 1 to Time 3.

**Table 3:** Consultation and prescribing data

	*Intervention arm,* n*=193*	*Control arm,* n*=88*	*95% CI* a
Total (and mean per patient) number of consultations^b^	200 (1.04)	85 (0.97)	−0.02, +0.63
Total (and mean per patient) number of rhinitis consultations^b^	55 (0.28)	29 (0.33)	−0.24, +0.08
Total (and mean per patient) number of consultations for other respiratory conditions^b^	27 (0.14)	8 (0.09)	−0.01, +0.22
Total (and mean per patient) number of prescriptions^b^	557 (2.89)	197 (2.24)	+0.08, +2.15
Total (and mean per patient) number of prescriptions for rhinitis^b^	406 (2.10)	140 (1.59)	−0.10, +0.12

Abbreviation: CI, confidence interval.

a 95% CI for difference in mean between intervention and control groups.

b Cumulative total for all patients from the date they were seen for the trial consultation to 31 August 2009 or 2010.

## Main topics covered in the seasonal allergic rhinitis training workshop

- History taking
- Diagnosis
- Treatment and management of allergic airways
- Identification and management of relevant co-morbidities
- Compliance and nasal spray device technique
- Organisational issues

## Notes

SW is an Associate editor of, and AS is Joint Editor-in-Chief of, the PCRJ. Neither were involved in the editorial review of, nor the decision to publish, this article. All other authors declare no conflict of interest.
